# Gut Microbiota as a Mediator of Dietary Salt Effects on Blood Pressure

**DOI:** 10.3390/ijms27104515

**Published:** 2026-05-18

**Authors:** Barbara J. H. Verhaar

**Affiliations:** 1 Department of Vascular Medicine, Amsterdam UMC, 1105 AZ Amsterdam, The Netherlands; b.j.verhaar@amsterdamumc.nl; Tel.: +31-020-566-9111; 2 Amsterdam Cardiovascular Sciences (ACS) Research Institute, 1105 AZ Amsterdam, The Netherlands; 3 Amsterdam Gastroenterology Endocrinology Metabolism (AGEM) Research Institute, 1105 AZ Amsterdam, The Netherlands

**Keywords:** dietary salt, gut microbiome, hypertension, salt sensitivity, osmoadaptation

## Abstract

Dietary sodium excess is a primary driver of hypertension, yet individuals differ markedly in their blood pressure response to salt. This variation, termed salt sensitivity, cannot currently be predicted from clinical variables alone. This review examines three aspects of salt-gut physiology: intestinal sodium handling, salt-induced changes in gut microbiome composition, and microbiota-mediated effects on immune function, metabolite production, and gut barrier integrity. The intestine absorbs dietary sodium through regulated transporters whose activity adapts to luminal and hormonal conditions, making the gut a key regulator of sodium balance. High salt intake consistently alters gut microbiota composition in animal models, most reproducibly depleting *Lactobacillus* species, with variable effects on overall diversity. These compositional shifts, supported by human data, activate intestinal Th17 cells and deplete short-chain fatty acid producers, contributing to systemic inflammation and elevated blood pressure. The presence of inducible osmoadaptation responses varies substantially across microbes, though activation under dietary sodium conditions has not been demonstrated *in vivo*. If salt-driven microbial changes contribute causally to hypertension, microbiota-targeted interventions could complement sodium restriction in patients for whom long-term dietary adherence is poor. Controlled sodium intervention studies in animals and humans are needed to establish whether such a causal contribution exists.

## 1. Introduction

Hypertension is the leading preventable cause of cardiovascular disease worldwide [1]. Excess dietary sodium is a primary driver: global intake consistently exceeds the World Health Organisation (WHO) recommendation of under 5 g of salt per day [2,3], yet adherence to reduction guidelines remains poor, and debate persists over whether universal restriction or targeted interventions offer greater benefit [4].

The link between high sodium intake and elevated blood pressure is well-established [5], but the underlying biology extends well beyond renal sodium handling. Tissue sodium storage, immune cell activation, and vascular tone all shape individual salt sensitivity [6,7]. More recently, the gut microbiome has emerged as another determinant: bacterial communities differ in their capacity to tolerate and metabolise salt, and high-salt diets alter microbial composition in ways that may influence immune and cardiovascular function [8,9,10,11,12]. Much of this evidence on gut microbiota composition and cardiovascular health has relied on 16S rRNA sequencing, which profiles community composition but cannot resolve gene content or functional capacity. Shotgun metagenomic sequencing is now increasingly used and enables systematic profiling of osmoadaptation gene carriage across gut commensals.

This review addresses three questions: how the intestine handles sodium, how high salt intake reshapes the gut microbiome, and how microbial changes in turn modify the cardiovascular response to salt. Together, these mechanisms may help explain interindividual variations in salt sensitivity and point toward new targets for prevention and treatment.

## 2. Salt and Human Physiology

### 2.1. Dietary Sodium Consumption

For humans, sodium is an essential mineral that must be obtained from the diet. When sodium levels drop, neurons in the hypothalamus sense the deficit and generate a specific craving for salt [13]. In rodents, this response is mediated primarily by epithelial sodium channel (ENaC)-expressing taste receptor cells. In humans, ENaC is one of the contributors to salt taste [14]. ENaC also drives sodium absorption in the gut and kidney, linking taste to systemic sodium handling [15].

Dietary salt is consumed predominantly as sodium chloride, and sodium is essential for every process dependent of Na^+^/K^+^-ATPase activity, including nerve signalling and muscle contraction. In healthy adults, extracellular sodium is tightly maintained between 135 and 145 mmol/L through renal, vascular, and hormonal regulation [16]. Deviation from this range in either direction has clinical consequences: hyponatraemia causes weakness and cognitive impairment; hypernatraemia causes neuromuscular irritability [17,18].

The elaborateness of sodium-retaining mechanisms (spanning the kidney, gut, and endocrine system) points to an evolutionary history of chronic scarcity [19,20], consistent with sodium intakes below 1 g per day in traditionally living populations such as the Yanomami [21]. In modern diets, the majority of dietary sodium comes not from discretionary salt use but from processed foods [22], which limits the effectiveness of individual dietary restriction. Average intake in Western populations reaches 9–12 g of salt per day, well above the WHO recommendation of less than 5 g based on cardiovascular risk reduction [2,3]. As of 2022, no country had achieved the WHO sodium reduction target, despite widespread policy commitments [3].

### 2.2. The Gut as First Regulatory Barrier for Sodium

Dietary sodium is absorbed efficiently along the gastrointestinal tract, with regional differences in transport mechanisms and regulation. In the small intestine, sodium uptake is predominantly active and transcellular, tightly coupled to nutrient absorption. After a meal, sodium is absorbed together with glucose via sodium-glucose cotransporter 1 (SGLT1), whose activity scales with how much glucose is present in the gut lumen [23]. Between meals, a second transporter, sodium-hydrogen exchanger 3 (NHE3), takes over: it exchanges sodium for hydrogen in a process that does not depend on nutrient availability [24,25]. Both routes, on the apical side of the intestinal cells, are powered by the activity of Na^+^/K^+^-ATPase on the basolateral side, which maintains the electrochemical gradient that drives uptake. The balance between the two shifts with feeding state and with intracellular signalling, giving the small intestine considerable flexibility in how it handles sodium across the day.

In the distal small intestine and proximal colon, sodium absorption increasingly occurs via electroneutral NaCl uptake, mediated by the coordinated activity of NHE3 and the chloride–bicarbonate exchanger DRA (SLC26A3) [26]. This pathway is the dominant mechanism during fasting conditions and is highly sensitive to intracellular signalling. Increases in cyclic adenosine monophosphate (cAMP), cyclic guanosine monophosphate (cGMP), or intracellular Ca^2+^ reduce electroneutral NaCl absorption, providing an important mechanism by which neural, hormonal, and paracrine signals can rapidly modulate intestinal sodium uptake [27].

The colon, although handling a smaller absolute sodium load, plays a key regulatory role due to its high efficiency and reserve absorptive capacity. In the distal colon, sodium absorption becomes predominantly electrogenic and is mediated by ENaC. Aldosterone regulates ENaC through transcriptional and posttranslational mechanisms while coordinately upregulating basolateral Na^+^/K^+^-ATPase, enabling efficient sodium retention independent of nutrient transport [28,29].

In addition to transcellular pathways, paracellular sodium transport contributes throughout the intestine in a region-dependent manner, determined by local electrochemical gradients and epithelial tight junction permeability, and is most prominent in the proximal small intestine [30]. Together, these mechanisms underscore that the intestine is not a passive conduit, but an actively regulated and adaptable organ.

### 2.3. Sodium Handling by the Kidneys

The kidney is central to long-term regulation of sodium balance and blood pressure. Water homeostasis can be corrected rapidly through plasma osmolality sensing and vasopressin (ADH) release, whereas sodium balance is regulated more slowly and determines extracellular volume [31]. Despite wide variation in dietary intake, plasma sodium remains stable because ~99% of filtered sodium is reabsorbed under physiological conditions, meaning that small changes in tubular handling have large physiological effects. Most sodium reabsorption occurs in the proximal tubule via sodium-coupled transporters and the Na^+^/H^+^ exchanger NHE3, while the distal nephron provides hormonally regulated finetuning [32,33]. Here, the Na^+^/Cl^−^ cotransporter (NCC) and ENaC determine final sodium excretion under control of the renin-angiotensin-aldosterone (RAS) system [31,34]. Transcellular sodium uptake is driven by the basolateral Na^+^/K^+^-ATPase, a conserved mechanism shared with intestinal epithelia [35]. Impaired downregulation of distal sodium reabsorption during high salt intake limits natriuresis and contributes to salt-sensitive increases in blood pressure [4,36].

### 2.4. Dietary Sodium and Blood Pressure Regulation

Dietary sodium is a primary determinant of extracellular fluid volume and blood pressure [37]. Acute increases in sodium intake expand plasma volume and raise cardiac output, but healthy individuals compensate through pressure natriuresis and suppression of RAS, restoring sodium balance without a sustained pressure rise [38,39]. At the population level, higher sodium intake associates consistently with higher blood pressure and a steeper age-related rise [2,40]. Indigenous populations with very low sodium intake, such as the Yanomami, show minimal age-related pressure increase [20], though these observations may be confounded by broader dietary and lifestyle differences. Direct evidence that sodium reduction prevents major cardiovascular events remains limited; most of the inferred benefit runs through modest blood pressure reductions rather than demonstrated effects on hard outcomes [41,42]. This limitation reflects the absence of long-term randomised controlled trials with pre-specified cardiovascular endpoints, the modest and heterogeneous effect sizes observed across studies, and continued reliance on observational evidence for extrapolating cardiovascular benefit beyond blood pressure.

Blood pressure responses to sodium intake vary considerably between individuals [43,44]. Many show little or no change across a wide range of intake–salt resistance–through coordinated renal, vascular, and neurohormonal buffering [43,45]. Others show a clear pressure rise with higher sodium exposure [43,44]. Salt sensitivity is better understood as the upper end of a continuous distribution of blood pressure responses than as a discrete category; classification into salt-sensitive or salt-resistant groups depends heavily on experimental protocol, loading strategy, and cut-off choice [4,46,47]. Salt sensitivity is also not a fixed trait: individuals shift along this spectrum with ageing, declining kidney function, and worsening vascular and metabolic health [44]. This variability has been attributed to differences in renal sodium handling and vascular responsiveness [38,45].

The gastrointestinal tract is where dietary sodium first enters the body, yet its role in sodium balance is often underappreciated [48]. Intestinal absorption is not passive: it is mediated by regulated transporters and channels whose activity varies between individuals and adapts to dietary conditions [32]. Intestinal sodium handling also interacts with local immune and microbial processes, influencing how much sodium reaches the circulation and how the host responds [9,49,50]. The gut is therefore an upstream control point in blood pressure regulation that may help explain interindividual differences in salt sensitivity that renal models alone do not account for.

## 3. Sodium and the Gut Microbiome

Evidence on the impact of dietary salt on the gut microbiome originates from in vitro studies, animal experiments, population-based studies, and two intervention trials.

### 3.1. The Effect of Sodium on Microbes

High extracellular sodium draws water out of bacterial cells by osmosis, reducing turgor pressure and impairing growth; at sufficiently high concentrations, it is lethal [51]. At low concentrations, however, Na^+^ is required by virtually all bacteria for ion homeostasis, pH regulation, and solute transport. Most species keep intracellular Na^+^ low through Na^+^/H^+^ antiporters, while Na^+^-coupled symporters drive uptake of amino acids and sugars [52,53].

A subset of bacteria additionally uses sodium as a motive force [54]. Most cellular processes are powered by an electrochemical gradient across the cytoplasmic membrane: in the majority of species, the proton motive force, generated when respiratory enzymes pump protons out of the cell to create a combined electrical and chemical gradient [53]. The return flow of protons drives ATP synthesis, nutrient uptake, and flagellar rotation [55]. Some bacteria, however, integrate sodium directly into primary energy conservation, including marine and halophilic species such as *Vibrio cholerae*, alkaliphiles, and strictly anaerobic fermenters and acetogens such as *Acetobacterium woodii* [53,54,56]. In these organisms, respiratory or fermentative complexes expel Na^+^ instead of, or in addition to, protons, generating a sodium motive force that powers Na^+^-dependent ATP synthases, transporters, and flagella [53,55]. This is advantageous in alkaline, saline, or energy-limited anaerobic environments, where maintaining steep proton gradients is energetically costly [54,57]. That proton and sodium bioenergetic circuits coexist, and in some species are interchangeable, suggests sodium gradients are a conserved alternative to proton-based energy conservation, particularly in marine environments where Na^+^ is abundant [53,54].

Halophiles are microorganisms adapted to high-salt environments, from salt lakes to marine brines, and have remodelled their proteomes, membranes, and bioenergetics accordingly [58]. Two main strategies underlie this adaptation. In “salt-in” strategy, K^+^ and Cl^−^ are accumulated intracellularly, which requires proteins with acidic surfaces stable under high ionic strength [58]. Most moderately halophilic bacteria instead keep intracellular Na^+^ low using compatible solutes—organic osmolytes such as glycine betaine or ectoine—that raise intracellular osmolality without disrupting protein function [59].

**Figure 1:**
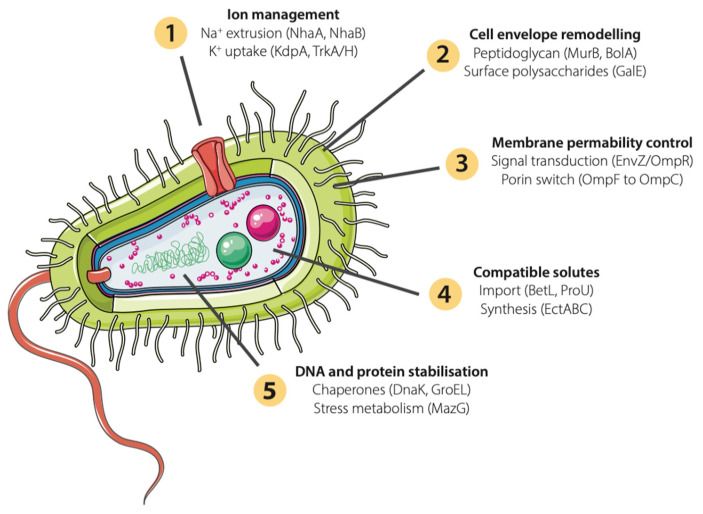
Osmoadaptation strategies in gut bacteria in response to elevated luminal sodium. Schematic of a Gram-negative bacterium illustrating five categories of osmotic stress response. Categories 2 and 3 include mechanisms specific to Gram-negative species. Whether these responses are activated under osmotic conditions generated by dietary sodium intake has not been demonstrated *in vivo*.

Many gut bacteria encode inducible osmotic stress responses, although their relevance in gut bacteria is largely uncharacterised. Where responses have been studied, they involve a combination of ion extrusion, compatible solute accumulation, and structural remodelling (Figure 1 and Table 1). The Na^+^/H^+^ antiporters NhaA and NhaB extrude sodium while the potassium uptake systems KdpA and TrkA/H restore turgor [60,61,62,63]. Compatible solutes are imported via BetL or the high-affinity ABC transporter ProU, or synthesised *de novo* through the EctABC pathway [64,65,66,67,68]. Some species also remodel their cell envelope: MurB and BolA reinforce peptidoglycan integrity, and in Gram-negative bacteria the EnvZ/OmpR two-component system shifts outer membrane composition from the large-pore porin OmpF to the small-pore OmpC, reducing passive ion influx [69,70,71,72,73].

Osmotolerance varies substantially across gut commensals. Ng et al. profiled 92 human gut strains across an NaCl gradient and found Lactobacillaceae and Enterococcaceae to be broadly osmotolerant, while Bacteroidaceae, most Bifidobacteriaceae, Lachnospiraceae, and *Akkermansia muciniphila* were osmosensitive. The largest heterogeneity was found within the Lactobacillaceae, with some strains not growing in high osmolality while others could. Generally, strains with certain (broad) annotations of osmotic stress genes showed more growth at higher osmolality [11]. Whether gut commensals induce these responses under the osmotic conditions created by dietary sodium intake has not been demonstrated *in vivo*.

### 3.2. Mice Studies on Salt and the Gut Microbiome

High-salt feeding frequently alters gut microbial composition in mice models and reduces alpha diversity [79,80,81]. The most reproducible finding is depletion of *Lactobacillus* species, with *Lactobacillus murinus* specifically identified as a salt-sensitive taxon in mice [9,79,80,81]. However, this pattern was not observed in wildling mice, which harbour a richer, wild-derived microbiota. These animals showed only minor compositional shifts under high-salt feeding, suggesting that the magnitude of salt-induced microbial shifts depends on the baseline diversity and composition of the gut microbiome [80].

Several studies also report decreases in the Firmicutes to Bacteroidetes ratio under high-salt conditions [79,81]. Beyond Lactobacilli, high-salt feeding in conventional laboratory mice has been associated with depletion of *Bifidobacterium*, *Blautia*, *Faecalibacterium*, and *Turicibacter* [79,80]. High-salt feeding in Wilck et al. additionally reduced *Akkermansia muciniphila*, in line with another mice study [9,79]. An in vitro study also showed that an *Akkermansia* strain was osmosensitive, in line with the observation in wildling mice that *Akkermansia* decreases with a high-salt diet [11,80]. These could be strain-dependent or community-dependent effects. Dietary sodium loading and impaired epithelial sodium reabsorption (NHE3 knockout) produce broadly similar compositional shifts [82], including a relative increase in Bacteroidetes species.

That osmotic conditions in the gut lumen act as a selective pressure on community composition has been demonstrated directly in mice: raising luminal osmolality from ~533 to ~810 mOsm/kg with the osmolyte polyethylene glycol (PEG) caused reproducible extinction of osmosensitive microbes. In surviving strains, there was an enrichment of genes involved in osmotic and dehydration stress and membrane transport (profiled with MG-RAST) [12]. However, the relative contributions of osmolality, ionic composition (sodium chloride versus PEG), baseline microbiome, and the specific genes responsible for the osmoadaptation remain to be elucidated.

### 3.3. Human Studies on Salt and the Gut Microbiome

Human evidence for an effect of dietary salt on gut microbiota composition derives from population studies and two intervention trials. In the population-based Shika Study, the association between sodium intake and hypertension prevalence differed by gut microbiome enterotype, pointing to effect modification by gut microbiota [83]. Two population studies located in Kazakhstan and Ghana found associations between higher sodium intake in urban settings and reduced microbiome diversity, though the effects cannot be disentangled from a broader dietary transition [84,85]. A population study in 2833 Chinese adults further showed that energy-adjusted sodium intake (sodium density) was associated with distinct microbial taxa and plasma metabolites, and that microbiome composition partially predicted habitual sodium exposure [86].

The foundational human study in this field was a 14-day salt challenge in 12 healthy men, in whom habitual intake was supplemented with 6 g NaCl per day (total ~13.8 g/day) using slow-release tablets, with shotgun metagenomics performed on stool at baseline and day 14 [9]. However, *Lactobacillus* is not a dominant member of the adult human gut microbiome—only five of the 12 participants had any detectable *Lactobacillus* species at baseline. Of the ten *Lactobacillus* populations present at baseline, nine were undetectable after the challenge. Yet, several participants simultaneously gained new *Lactobacillus* species not present at baseline, which the authors attributed to dietary intake of *Lactobacillus*-containing foods rather than salt-driven colonisation, as no dietary restrictions were imposed. Survival analysis also showed an accelerated loss of non-*Lactobacillus* species, which were not individually identified. Blood pressure and circulating Th17 cells both increased over the challenge period [9]. Taken together, this trial suggests that a salt challenge can reduce the survival of specific *Lactobacillus* species and raise blood pressure.

The largest dedicated intervention study to date, the MetaSalt trial, recruited 528 participants in a 23-day crossover design comprising a 3-day baseline observation on usual diet, a 10-day low-salt phase (3 g/day), and a 10-day high-salt phase (18 g/day), with shotgun metagenomics and plasma metabolomics at each phase [10]. Alpha diversity increased on the low-salt diet and decreased on the high-salt diet, although overall community composition (beta diversity) did not shift significantly across interventions. High salt significantly changed the abundance of 85 microbial species at Bonferroni-corrected thresholds, of which 79 were depleted. The most strongly depleted species were *Subdoligranulum variabile* and *Gemmiger formicilis*, alongside *Pseudoflavonifractor capillosus*, *Intestinimonas butyriciproducens*, *Anaerotruncus colihominis*, and a *Faecalibacterium* species; *Akkermansia muciniphila* was also depleted. Changes in 22 species were associated with salt-sensitive blood pressure. A gut microbiota-acylcarnitine network was identified as a potential mediating pathway, with isovalerylcarnitine as the core metabolite inversely associated with salt-sensitive blood pressure [10].

Both trials have their limitations. The trial of Wilck et al. enrolled only 12 healthy men, limiting generalisability across sexes. Sodium supplementation was administered on top of uncontrolled and unmeasured habitual intake, introducing variability of sodium exposure across participants. The MetaSalt trial administered salt as food rather than capsules, meaning sodium intake was altered alongside other dietary variables; the fixed-sequence crossover without randomisation of diet order and without a wash-out period between phases further introduces potential carry-over effects. These two trials focused primarily on microbiota composition: the downstream effects on microbial pathway activity and host physiology remain incompletely characterised. Larger trials are needed, incorporating functional metagenomics alongside compositional profiling across diverse populations and salt exposure ranges. Another remaining gap is the physiological range of colonic sodium concentrations in humans, and how sodium intake affects local concentrations and the variance thereof in the colon, where most of the microbial diversity can be found. More broadly, the human evidence base in this field remains thin: only two intervention trials have been published, both with methodological constraints that limit causal inference. Population studies establish associations but cannot disentangle sodium from correlated dietary exposures. Effect sizes in the intervention data are modest, and no study has yet demonstrated that salt-induced microbial changes mediate a measurable blood pressure effect independently of direct physiological sodium actions.

## 4. Mechanistic Pathways Linking Salt, Microbes, and Cardiometabolic Disease

Dietary salt intake may contribute to hypertension not only via host physiology but also through microbiota-mediated immune and metabolic pathways. The following sections summarise the evidence on salt-induced microbial immune modulation, changes in microbial metabolite production, and microbe-mediated effects of salt on gut barrier integrity.

### 4.1. Microbe-Immune Interactions

Hypertension has a clear inflammatory component, involving both the innate and adaptive immune system. Monocytes and macrophages accumulate in the kidney, vasculature, and perivascular adipose tissue, where they sustain local inflammation and impair sodium handling [87]. Those in the interstitium and the skin additionally regulate lymphatic sodium clearance via tonicity-responsive enhancer binding protein (TonEBP)/vascular endothelial growth factor C (VEGF-C) signalling [6,88]. Additionally, the Th17/Treg balance is central to hypertension [87]. Th17 cells promote renal sodium retention, vascular oxidative stress, and endothelial dysfunction, while Tregs suppress effector T cell activation and maintain vascular homeostasis [87,89]. CD8^+^ cytotoxic T cells also contribute to hypertension [87]. In angiotensin-induced hypertension in mice, infiltrating CD8^+^ T cells are the main source of interferon gamma (IFNγ) in the kidney and vasculature. IFNγ drives upregulation of distal tubular sodium cotransporters and impairs endothelial nitric oxide bioavailability [89].

Dietary salt drives immune activation through two routes: directly via effects on the ionic microenvironment of immune cells and indirectly through the gut microbiome. High intraluminal sodium activates mucosal dendritic cells directly, independent of microbes. Sodium enters dendritic cells via ENaC, activates NADPH oxidase, and promotes lipid peroxidation, generating isolevuglandins (IsoLGs) that adduct to intracellular proteins [90]. These IsoLG-adducted proteins act as neoantigens and drive T-cell activation and IFN-γ and interleukin-17A (IL-17A) production [91,92].

The indirect effects of salt could act through the depletion of immunomodulatory bacteria. High-salt conditions deplete intestinal *Lactobacillus* species in mice, expand Th17 numbers in the lamina propria and spleen, and raise blood pressure. Oral recolonisation normalises Th17 frequencies and attenuates hypertension [9]. The same study showed that short-term salt loading in healthy humans reduced intestinal *Lactobacillus* survival, increased circulating Th17 cells, and raised blood pressure [9]. Many bacteria depleted under high-salt conditions have established anti-inflammatory roles in other disease contexts: *Faecalibacterium prausnitzii* has been shown to promote Treg differentiation and suppress NF-κB-driven cytokine production, while *Akkermansia muciniphila* contributes to epithelial barrier integrity and limits lipopolysaccharide (LPS) translocation [93,94]. Whether their depletion by dietary salt contributes to mucosal immune activation in hypertension has not been directly tested.

### 4.2. Metabolite Production

Gut bacteria produce metabolites that modulate vascular physiology directly, and high dietary salt perturbs their production. Short-chain fatty acids (SCFA) are produced by colonic fermentation of otherwise indigestible dietary fibres by anaerobic bacteria, predominantly members of Lachnospiraceae and Ruminococcaceae. The most abundant SCFA are butyrate, propionate, and acetate [95,96]. In rodents, depletion of SCFA-producing bacteria under high-salt conditions reduces luminal butyrate [81] and propionate availability [97]. These metabolites act through two mechanisms. First, they activate G protein coupled receptor 41 (GPR41) and GPR43, expressed on vascular smooth muscle cells, endothelium, and circulating immune cells, to promote vasodilation [98,99,100]. GPR41 knockout mice show isolated systolic hypertension [99], while GPR41/43 double knockout mice under angiotensin II stimulation show higher systolic blood pressure, perivascular fibrosis, and a cardiorenal phenotype [100]. In a cohort of ~276,000 UK Biobank participants, genetic variants associated with higher GPR41 and GPR43 expression were associated with lower hypertension prevalence [100]. Second, butyrate directly inhibits histone deacetylase (HDACs) in endothelial cells and vascular smooth muscle cells, suppressing LPS- and tumour necrosis factor α (TNFα)-induced interleukin (IL)-8 production, vascular cell adhesion molecule-1 (VCAM-1) expression, and monocyte adhesion to endothelium [101]. Butyrate additionally inhibited vascular smooth muscle cell proliferation through chromatin remodelling [102].

Whether reduced SCFA availability under high salt translates to a clinically meaningful blood pressure effect in humans is uncertain. A randomised placebo-controlled trial of oral sodium butyrate (4 g/day, 4 weeks) in 23 patients with mild hypertension found that butyrate increased daytime systolic blood pressure by 9.6 mmHg and diastolic blood pressure by 5.1 mmHg, with no mechanistic explanation in terms of renin-aldosterone activation, sympathetic tone, or immunophenotype [103]. In contrast, colonic delivery of acetate and butyrate via acetylated and butyrylated high-amylose maize starch reduced 24-h systolic blood pressure by ~6 mmHg in untreated hypertensive patients [104], suggesting that the cardiovascular effect of butyrate depends on the delivery route and the extent of systemic absorption.

Gut bacteria are required for secondary bile acid synthesis; germ-free mice lack secondary bile acids, and antibiotic-mediated disruption of the intestinal microbiota reduces bile acid levels [105]. Secondary bile acids are agonists for Takeda G protein-coupled receptor 5 (TGR5), which in vascular endothelial cells induces endothelial nitric oxide synthase activity (eNOS), reduces monocyte adhesion, and suppresses NF-κB activation [106,107]. FXR and TGR5 are expressed in the kidney and gut, where they have been implicated in sodium and water homeostasis; bile acids can both activate and inhibit ENaC, though the mechanistic links to salt-sensitive hypertension remain to be established [105]. In Dahl salt-sensitive rats, a high-salt diet increased four bile acid species whose levels correlated positively with circulating endothelin-1 and TNFα [108]. These data suggest that high dietary salt affects the bile acid pool, which might influence vascular inflammation via FXR/TGR5 signalling.

### 4.3. Gut Barrier Integrity

High dietary salt disrupts epithelial barrier integrity through both direct osmotic effects on colonocytes and microbiota-dependent mechanisms. In mice fed 2% NaCl in drinking water for eight weeks, Hu et al. demonstrated reduced colonic expression of the barrier-forming tight junction proteins zona occludens-1 and occludin, accompanied by increased gut permeability measured with a fluorescent tracer (FITC-dextran) passage and elevated plasma endotoxin [109]. These effects were rescued by the addition of an antibiotic to the treatment, suggesting the gut microbiota mediated these effects. Miranda et al. similarly showed that the exacerbation of experimental colitis by a high salt diet was absent in germ-free mice, further supporting the role of the gut microbiota as mediators of barrier vulnerability [81]. The human evidence on the effects of salt on the gut barrier is, however, very limited.

The gut barrier effects of salt can be mediated by SCFA, and have downstream effects on the immune system. As described in Section 4.2, high-salt induced microbial shifts may reduce butyrate availability. Since butyrate is the primary energy substrate for colonocytes and drives tight junction assembly and mucus production, its loss weakens the gut barrier [110]. The downstream consequence of increased permeability is the translocation of microbial products (predominantly LPS) into the portal and systemic circulation. Circulating LPS activates toll-like receptor 4 (TLR4) on endothelial cells and macrophages, sustaining low-grade inflammation and impairing endothelial function [111]. Cytokines elevated downstream of the Th17 expansion described in Section 4.1 further reduce tight junction protein expression, further reinforcing the loop of gut barrier disruption and vascular inflammation [112].

## 5. Future Perspectives

The evidence reviewed here has important limitations. Human intervention data rest on two trials with design limitations, as discussed above, and population studies cannot disentangle the impact of sodium from broader dietary transitions. The mechanistic chain from salt-induced microbial shifts to blood pressure has not been demonstrated in humans, and whether baseline microbiome composition predicts individual blood pressure responses to sodium has not been tested in an adequately powered trial. No study has shown that gut bacteria activate osmoadaptation responses under the luminal sodium concentrations generated by dietary intake. Closing these gaps will require controlled sodium challenge studies in animals and humans, with metagenomics and transcriptomics to establish whether osmoadaptation responses are induced under physiologically relevant sodium conditions.

Accurate sodium exposure assessment is a prerequisite for future studies on salt and the gut microbiome. Urinary 24-h collections are a good reflection of excreted sodium but burdensome, and nutritional diaries systematically underreport consumption [113]. Disentangling dietary sodium from correlating exposures is nearly impossible in dietary sodium interventions. In Western diets, high sodium intake correlates with the consumption of processed foods, which are also high in fat and low in potassium and fibre. Dietary fibre is particularly relevant, since it not only stimulates the growth of SCFA-producing microbes, but also has ion-binding capacity that may reduce luminal sodium availability [114]. Sodium administered by capsules increases intake in a controlled and stable manner without altering other dietary variables, making it better suited to investigate sodium-specific effects. A combination of repeated 24-h urine collection, dietary self-report, faecal salinity measurement, and skin sodium quantification [115] would capture the full range of sodium handling across individuals and time.

Sex has received little attention in salt-microbiome research, despite being relevant to the gut microbiome composition, blood pressure regulation, and the immune system. Women show a steeper age-related blood pressure rise from the third decade onward than men, independent of cardiovascular risk factors [116], and plasma metabolite profiles associated with blood pressure differ substantially between sexes, with several gut microbiota-associated metabolites among the top predictors [117]. Oestrogen promotes Foxp3+ Treg expansion and attenuates Th17 activation [118,119], which may blunt the salt-induced Th17-mediated blood pressure response in premenopausal women and explain these divergent trajectories. Sex differences in gut microbiome composition and functional pathways have also been documented in large population-based data [120], warranting sex stratification in future microbiome-salt intervention trials.

The mechanistic framework reviewed here has potential clinical implications. If microbes or microbial pathways prove sufficiently sensitive and reproducible, they could index habitual dietary sodium exposure, addressing a longstanding gap in nutritional epidemiology where self-reported intake is notoriously unreliable. If salt-driven microbial changes contribute causally to hypertension, microbiota-targeted interventions could complement sodium restriction, particularly in patients for whom long-term adherence to dietary change is poor [4,121], though designing such interventions is premature until the causal contribution of microbial shifts to blood pressure is established in humans. Microbiome profiling as a stratification tool to identify patients most likely to respond to sodium restriction remains a longer-term possibility.

## 6. Conclusions

**Figure 2:**
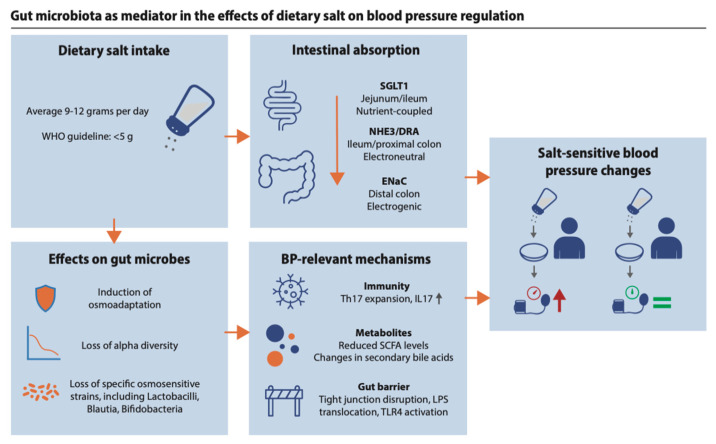
Gut microbiota as mediator in the effects of dietary salt on blood pressure regulation. Dietary salt intake in Western populations averages 9–12 g per day, well above the WHO recommendation of less than 5 g. Sodium is absorbed in the gut through transporters with distinct regional distribution: SGLT1 in the jejunum and ileum (nutrient-coupled), NHE3/DRA in the ileum and proximal colon (electroneutral), and ENaC in the distal colon (electrogenic, aldosterone-regulated). High luminal sodium concentrations alter gut microbial community structure, selecting for osmoadapted strains and depleting osmosensitive taxa including *Lactobacillus*, *Blautia*, and *Bifidobacterium* species, with a reduction in alpha diversity. These changes may affect blood pressure through three pathways: expansion of intestinal Th17 cells and IL-17 production; reduced short-chain fatty acid levels and altered secondary bile acid metabolism; and impaired gut barrier integrity, with tight junction disruption, LPS translocation, and TLR4 activation. Together these mechanisms contribute to salt-sensitive blood pressure elevation. BP, blood pressure; DRA, down-regulated in adenoma (SLC26A3); ENaC, epithelial sodium channel; IL-17, interleukin-17; LPS, lipopolysaccharide; NHE3, Na^+^/H^+^ exchanger 3; SCFA, short-chain fatty acids; SGLT1, sodium-glucose cotransporter 1; Th17, T helper 17 cell; TLR4, Toll-like receptor 4; WHO, World Health Organization.

High dietary salt depletes osmosensitive gut microbes, reduces SCFA availability, activates intestinal Th17 cells, and impairs barrier integrity (Figure 2). These changes may contribute to the interindividual variation in salt sensitivity that renal models alone do not explain. The evidence is largely associative. Causality has not been established in humans, and whether gut microbial osmoadaptation is activated under dietary sodium conditions remains untested. Controlled trials with pre-specified microbiome and cardiovascular endpoints, in diverse populations and across sexes, are the necessary next step. If causality can be established, microbiota-directed interventions could complement dietary sodium restriction in patients who cannot achieve sustained intake reduction.

## Figures and Tables

**Table 1:** Overview of genes associated with microbial osmoadaptation.

Gene	Name	Category	Description	Ref.
*nhaA*	Na^+^/H^+^ antiporter A	Ion homeostasis	Extrudes Na^+^ in exchange for H^+^; maintains low intracellular Na^+^; induced by elevated Na^+^ and osmotic stress	[60,61]
*nhaB*	Na^+^/H^+^ antiporter B	Ion homeostasis	Secondary Na^+^/H^+^ antiporter contributing to Na^+^ extrusion and pH homeostasis	[62]
*kdpA*	K^+^-translocating ATPase subunit	K^+^ uptake	High-affinity K^+^ uptake system mediating the rapid first-phase osmotic compensation response	[63]
*trkA/trkH*	K^+^ transporter Trk	K^+^ uptake	Constitutive low-affinity K^+^ uptake; contributes to immediate osmotic balancing	[63]
*betL*	Glycine betaine transporter	Compatible solute import	Imports glycine betaine from the environment; primary osmostress response in many Firmicutes	[64]
*proU*	Glycine betaine/proline ABC transporter	Compatible solute import	High-affinity ABC transporter for glycine betaine and proline; strongly induced under osmotic stress	[65]
*ectA*	L-2,4-diaminobutyric acid acetyltransferase	Ectoine synthesis	First enzyme in the ectoine biosynthesis pathway	[66,67,68]
*ectB*	Diaminobutyric acid aminotransferase	Ectoine synthesis	Second enzyme in the ectoine biosynthesis pathway	[66,67,68]
*ectC*	Ectoine synthase	Ectoine synthesis	Converts N-acetyl-diaminobutyric acid to ectoine; final step in biosynthesis	[66,67,68]
*murB*	UDP-N-acetylenolpyruvylglucosamine reductase	Cell wall remodelling	Peptidoglycan biosynthesis enzyme upregulated under osmotic stress to reinforce the cell envelope	[69]
*bolA*	Morphoregulatory protein BolA	Cell wall remodelling	Regulates cell shape and peptidoglycan remodelling; induced under osmotic and other stresses	[70]
*galE*	UDP-glucose 4-epimerase	Surface polysaccharide remodelling	Interconverts UDP-glucose and UDP-galactose for LPS and exopolysaccharide biosynthesis; modifies surface carbohydrate structures to reduce ion flux	[69]
*ompC*	Outer membrane porin C	Membrane permeability	Small-pore porin induced at high osmolarity via EnvZ/OmpR; replaces OmpF to reduce membrane permeability to ions	[72]
*ompF*	Outer membrane porin F	Membrane permeability	Large-pore porin repressed at high osmolarity; shift from OmpF to OmpC reduces ion influx under osmotic stress	[72]
*envZ*	Osmosensor histidine kinase	Signal transduction	Membrane-spanning osmosensor; phosphorylates OmpR in response to osmotic changes	[73]
*ompR*	Transcriptional response regulator	Signal transduction	Phosphorylated by EnvZ; regulates *ompC*, *ompF*, and other osmotic stress genes	[73]
*rpoS*	RNA polymerase sigma factor σS	Stress signalling	Master regulator of the general stress response; controls a large osmotic stress regulon in enterobacteria and gut commensals	[74]
*dnaK*	Chaperone protein DnaK (Hsp70)	Protein quality control	Stabilises and refolds proteins misfolded under osmotic stress; part of the broader heat and osmotic stress response	[75,76]
*groEL*	Chaperonin GroEL (Hsp60)	Protein quality control	Assists refolding of stress-denatured proteins; co-induced with *dnaK* under osmotic stress	[76,77]
*mazG*	Nucleoside triphosphate pyrophosphohydrolase	Stress metabolism	Hydrolyses (p)ppGpp and other nucleotides during stress; links osmoadaptation to the stringent response and stress signalling	[69]
*osmC*	Osmotically inducible protein C	Oxidative stress protection	Induced under osmotic stress; provides protection against co-occurring oxidative stress	[78]

Genes associated with microbial osmoadaptation, grouped by functional category. Evidence is drawn predominantly from well-characterised model organisms; conservation and inducibility across gut microbes has not been systematically established. The genes in this table fall into three functional categories: ion transporters that adjust intracellular sodium and potassium concentrations, biosynthesis pathways for compatible solutes that balance osmotic pressure without disrupting protein function, and general stress-response regulators.

## Data Availability

No new data were created or analyzed in this study. Data sharing is not applicable to this article.

## Abbreviations

The following abbreviations are used in this manuscript:

ADH	Antidiuretic hormone (vasopressin)
ATP	Adenosine triphosphate
cAMP	Cyclic adenosine monophosphate
cGMP	Cyclic guanosine monophosphate
DRA	Down-regulated in adenoma (SLC26A3)
ENaC	Epithelial sodium channel
eNOS	Endothelial nitric oxide synthase
FITC	Fluorescein isothiocyanate
FXR	Farnesoid X receptor
GPR41	G-coupled protein receptor 41
GPR43	G-coupled protein receptor 43
HDAC	Histone deacetylase
IFNγ	Interferon gamma
IL-17A	Interleukin-17A
IsoLG	Isolevuglandin
LPS	Lipopolysaccharide
NADPH	Nicotinamide adenine dinucleotide phosphate
NCC	Na^+^/Cl^−^ cotransporter
NF-κB	Nuclear factor kappa light chain enhancer of activated B cells
NHE3	Na^+^/H^+^ exchanger 3
PEG	Polyethylene glycol
RAS	Renin-angiotensin system
SCFA	Short-chain fatty acids
SGLT1	Sodium-glucose cotransporter 1
TGR5	Takeda G protein-coupled receptor 5 (GPBAR1)
Th17	T helper 17 cell
TLR4	Toll-like receptor 4
TNFα	Tumour necrosis factor alpha
TonEBP	Tonicity-responsive enhancer binding protein
Treg	Regulatory T cell
VCAM-1	Vascular cell adhesion molecule-1
VEGF-C	Vascular endothelial growth factor C
WHO	World Health Organization
